# Geometrical Stabilities and Electronic Structures of Rh_5_ Nanoclusters on Rutile TiO_2_ (110) for Green Hydrogen Production

**DOI:** 10.3390/nano14020191

**Published:** 2024-01-15

**Authors:** Moteb Alotaibi

**Affiliations:** Department of Physics, College of Science and Humanities in Al-Kharj, Prince Sattam Bin Abdulaziz University, Al-Kharj 11942, Saudi Arabia; mot.alotaibi@psau.edu.sa

**Keywords:** Rh_5_ nanoclusters, TiO_2_ rutile (110), photocatalysis, green hydrogen production, DFT, oxygen vacancy, sustainable energy

## Abstract

Addressing the urgent need for sustainable energy sources, this study investigates the intricate relationship between rhodium (Rh_5_) nanoclusters and TiO_2_ rutile (110) surfaces, aiming to advance photocatalytic water splitting for green hydrogen production. Motivated by the imperative to transition from conventional fossil fuels, this study employs density functional theory (DFT) with DFT-D3 and HSE06 hybrid functionals to analyse the geometrical stabilities and electronic structures of Rh_5_ nanoclusters on TiO_2_ rutile (110). TiO_2_, a prominent photocatalyst, faces challenges such as limited visible light absorption, leading researchers to explore noble metals like Rh as cocatalysts. Our results show that bipyramidal Rh_5_ nanoclusters exhibit enhanced stability and charge transfer when adsorbed on TiO_2_ rutile (110) compared to trapezoidal configurations. The most stable adsorption induces the oxidation of the nanocluster, altering the electronic structure of TiO_2_. Extending the analysis to defective TiO_2_ surfaces, this study explores the impact of Rh_5_ nanoclusters on oxygen vacancy formation, revealing the stabilisation of TiO_2_ and increased oxygen vacancy formation energy. This theoretical exploration contributes insights into the potential of Rh_5_ nanoclusters as efficient cocatalysts for TiO_2_-based photocatalytic systems, laying the foundation for experimental validations and the rational design of highly efficient photocatalysts for sustainable hydrogen production. The observed effects on electronic structures and oxygen vacancy formation emphasize the complex interactions between Rh_5_ nanoclusters and the TiO_2_ surface, guiding future research in the quest for clean energy alternatives.

## 1. Introduction

Contemporary chemistry is characterized by a heightened focus on addressing global challenges related to energy production and environmental remediation. The pursuit of innovative energy alternatives, driven by the pressing need to move beyond conventional fossil fuels, has become a central theme in scientific research. This shift towards sustainable practices is not only motivated by environmental consciousness but also by stringent environmental regulations that highlight the necessity of adopting eco-friendly approaches. A pivotal contribution to this field is exemplified by the groundbreaking work of Fujishima and Honda, who showed the viability of photoelectrochemical water splitting using rutile TiO_2_ as an anode and a Pt wire cathode under a chemical bias [1]. Beyond its role in water splitting, TiO_2_, a prominent photocatalyst, has garnered significant attention for its applications in the decomposition of harmful organic materials [2,3,4,5]. This multifaceted functionality extends TiO_2_ photocatalysis to diverse environmental challenges, showcasing their effectiveness in both gaseous environments and solutions. This versatility aligns with the broader trend in contemporary chemistry, emphasizing interdisciplinary research approaches aimed at developing sustainable solutions for the interconnected issues of energy and the environment. The collaborative efforts of scientists and researchers across disciplines underscore the significance of integrated approaches in meeting the complex challenges of the modern era.

In photocatalytic water splitting [6], the role of TiO_2_ is pivotal but not without its challenges. TiO_2_ is commonly employed as a photocatalyst, and to intensify the efficiency of the hydrogen evolution reaction (HER), it is a prevalent practice to load suitable cocatalysts, often metallic in nature [7,8]. Upon photon excitation, TiO_2_ generates photoelectrons in its bulk region, underscoring the importance of cocatalysts that excel in two key aspects: (i) facilitating improved charge transfer over the interface from catalyst to the metal and (ii) ensuring fast H_2_ generation on the surface of metal [9,10]. However, TiO_2_ faces intrinsic challenges [11], including a band gap energy (Eg) of around 3.2 eV [12,13], that confines its light absorption primarily to ultraviolet (UV) wavelengths. Additionally, a notable concern arises from the significant degree of charge recombination for photo-generated charges in TiO_2_. In response to these challenges, researchers have implemented various strategies to broaden the application of TiO_2_ in photocatalysis. For instance, efforts have been made to integrate TiO_2_ with other semiconductor materials [14,15], a technique aimed at mitigating limitations associated with its band gap and extending its responsiveness to a broader spectrum of light. Furthermore, innovative approaches such as dye sensitization in solar cells have been explored [16], providing alternative avenues to enhance the performance of TiO_2_ in capturing and utilizing solar energy, fabricating with both metallic and non-metallic ions [17,18] and depositing noble metals [19]. These strategies exemplify the ongoing endeavours within the scientific community to overcome the inherent limitations of TiO_2_ and advance the field of photocatalysis for sustainable energy production.

Noble metals, such as Pd (palladium), Pt (platinum), and Rh (rhodium), have emerged as valuable catalysts in the realm of photocatalytic hydrogen evolution reaction (HER) due to their notable work functions and favourable Gibbs adsorption energies for hydrogen atoms [20,21]. These metals, with their distinctive properties, play a significant role in enhancing the efficiency of hydrogen evolution during photocatalysis. For example, the incorporation of Rh into TiO_2_ has been explored as a strategy to boost photoreactivity. Rh-doped TiO_2_ has demonstrated enhanced performance, a phenomenon attributed to the facilitated electron transfer between Rh and the TiO_2_ conduction band (CB) or valence band (VB). Studies have shown that TiO_2_ samples decorated with Rh exhibit superior activity compared to alternative modifications, emphasizing the effectiveness of noble metal doping in optimizing the photocatalytic properties of TiO_2_ [22,23]. This underscores the significance of exploring and understanding the synergistic effects between noble metals and semiconductor materials to further advance the development of effective photocatalysts for applications in sustainable energy.

Xing et al. [24] made notable contributions to the field of photocatalysis by employing a single-step approach to synthesise isolated metal atoms stably loaded on the TiO_2_ anatase (101) surface, thereby extending the concept of single atom catalysts to the domain of photocatalytic hydrogen production. This groundbreaking approach involves the deposition of single atoms of noble metals, including Pd, Pt, Rh, and Ru, uniformly on the TiO_2_ anatase (101) surface. The resulting catalysts exhibit remarkably improved photocatalytic performance, particularly in the context of hydrogen evolution. This innovative methodology opens new avenues for the design of highly efficient and stable photocatalysts. The work by Xing et al. underscores the importance of exploring diverse approaches to advance the understanding and application of single atom catalysts in photocatalysis. While Pt, Pd, Rh, and Ru nanoclusters have found practical applications [25,26], it is noteworthy that a comprehensive theoretical analysis of Rh nanocluster-loaded TiO_2_ rutile (110)-based photocatalytic systems is currently lacking. Further theoretical investigations into the unique properties and behaviours of these systems could provide valuable insights into their photocatalytic mechanisms and guide the development of advanced materials for sustainable energy applications.

In the current study, an examination of the pristine and reduced TiO_2_ rutile (110) surface loaded with Rh_5_ nanoclusters is conducted using density functional theory (DFT). Upon a reduction in the size of the Rh cluster, it exhibits a more substantial HOMO-LUMO gap relative to larger clusters of Rh [27]. The phenomenon of energy band discretisation in metal clusters is notably pronounced and size-dependent, resulting in the emergence of substantial band gaps in proximity to the Fermi level. These gaps often surpass one electronvolt (eV) and play a pivotal role in dictating the excitation–emission characteristics within clusters. Such a feature paves the way for the strategic design of metal clusters, capitalising on their distinctive luminescence properties across the ultraviolet-visible–infrared (UV-Vis-IR) spectrum for tailored optical applications [28]. Furthermore, an investigation into the low-index faces of rutile revealed that, in accordance with the composition found in natural rutile powder, the (110) face exhibits the highest stability, followed by the (100) and (101) faces in terms of their relative stability [29,30]. Therefore, the TiO_2_ rutile (110) surface was chosen in this study. The choice of employing the DFT-D3 method [31] is rooted in its effectiveness in characterising the adsorption behaviour of Rh nanoclusters on rutile TiO_2_. To further elucidate the electronic structure relevant to polaron formation on TiO_2_ surfaces [32,33], this study utilises the HSE06 hybrid functional, a theoretical framework developed by Heyd, Scuseria, and Ernzerhof [34,35]. This hybrid functional incorporates a fraction of exact exchange, improving the description of electronic properties compared to standard DFT methods. This article is organised as follows: Section 2 provides comprehensive details on the simulation methodologies employed, offering transparency and reproducibility in the research process. Section 3 is dedicated to presenting and discussing the outcomes of the simulations, shedding light on the interactions and behaviours observed in the system under investigation. It also delves into the concept of polaron and compares the obtained results with previous findings, contributing to the theoretical understanding of charge carriers on TiO_2_ surfaces. Finally, Section 4 outlines the principal results, providing a pragmatic perspective on their implications for the broader scientific community and applications in renewable energy.

## 2. Computational Details

To explore the electronic properties and charge density of Rh_5_ nanoclusters and understand their photon absorption capabilities, we employed the Vienna Ab initio Simulation Package (VASP 5.4.4) [36,37,38]. This allowed us to derive optimised geometries and electronic structures for both bare Rh_5_ nanoclusters and Rh_5_@TiO_2_. The simulations utilised the HSE06 hybrid exchange–correlation functional with periodic boundary conditions, encompassing short-range and long-range elements of the Perdew–Burke–Ernzerhof (PBE) exchange functional. The exchange–correlation component incorporates short-range Hartree–Fock (HF) exchange and a PBE correlation functional [34].

The interplay between valence electrons and the ion core was clarified through the utilisation of the projector-augmented wave (PAW) approach. [39,40], employing PAW-PBE [41] pseudopotentials. Valence electrons from Ti (3s, 3p, 4s, 3d), O (2s, 2p), and Rh (4d, 5s) atomic orbitals were considered. To rectify the self-interaction error and ensure accurate predictions of polaronic states and the band gap of TiO_2_, we incorporated the generalised-gradient approximation (GGA) with a Hubbard parameter (U) [42]. The assigned U value for the titanium (3d) state study was 4.2 eV, as documented in the literature [43,44]. Spin-polarized Perdew–Burke–Ernzerhof (PBE) with the Becke–Jonson (BJ) damping function, following Grimmme’s technique [31], was applied for van der Waals (vdW) corrections, selected for its accuracy in predicting the adsorption energy of metal oxide materials [45].

To model the pristine rutile TiO_2_ (110) surface, we constructed unit cells with dimensions of 12 Å × 13 Å, consisting of four O-Ti-O trilayers. A 20 Å vacuum layer was added above the surface. For individual Rh_5_ nanoclusters without any interaction with periodic images, we utilised large supercells (30 × 30 × 30 Å^3^). A k-point mesh was employed, adhering to the Monkhorst–Pack scheme [46], wherein all simulations were conducted utilising a singular k-point value, employing a fixed planewaves basis set of 500 eV. A Gaussian smearing parameter of 0.05 eV was applied for band occupation due to the substantial supercell used in the tetrahedron. To achieve self-consistent electronic minimisation, we set a convergence threshold of 10^−^^4^ eV. The convergence criterion of 10^−^^4^ eV was chosen based on common practices in computational studies [47,48,49,50,51]. This criterion ensures adequate precision in the calculations while balancing computational resources, and all modelled structures underwent relaxation with a force threshold value of 0.02 eV/Å.

The stability of catalysts during chemical reactions poses a crucial challenge for practical applications. Consequently, we computed the adsorption energy ($E_{ads}$) of the Rh_5_ nanocluster to assess its stability during adsorption, as per the following formula:

(1) $$E_{ads}=E_{tot}-E_{TiO2}-E_{Rh5}$$

Here, $E_{tot}$ is the total energy of the whole system, $E_{TiO2}$ is the total energy of TiO_2_, and $E_{Rh5}$ is the total energy of Rh_5_ nanoclusters. In the context of defects, the oxygen vacancy ($E_{Vo}$) formation energy was determined through the following formula:

(2) $$E_{Vo}=E_{surface+Vo}+\frac{1}{2}E_{O2}-E_{surface}$$

This involves $E_{surface+Vo}$, representing the total energy of the reduced TiO_2_, $E_{O2}$ denoting the total energy of free oxygen in the gas phase, and $E_{surface}$ representing the total energy of the perfect TiO_2_. Both adsorption and formation energies were assessed using GGA + U calculations. The construction and visualisation of all structures presented in this study were performed using (VESTA 3.5.8) software [52].

## 3. Results and Discussion

### 3.1. Isolated Rh_5_ Nanoclusters

**Figure 1:**
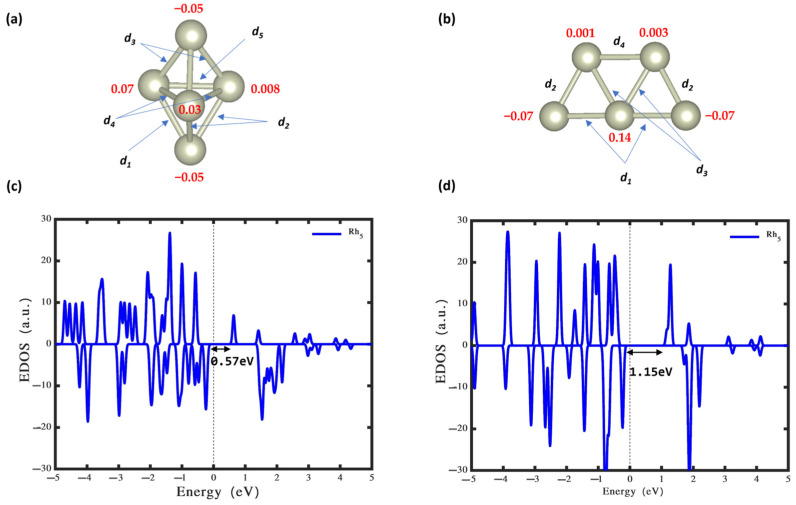
(**a**) Bipyramidal and (**b**) trapezoidal Rh_5_ nanoclusters. Red numbers show the amount of electron on each atom. *d*_1_–*d*_5_ represent the Rh-Rh bond lengths. The relevant values are provided in Table 1. (**c**) Density of states of bipyramidal Rh_5_. (**d**) Density of states trapezoidal Rh_5_.

Figure 1a and Figure 1b illustrate the optimised structures of Rh_5_ nanoclusters in the gas phase, showcasing bipyramidal and trapezoidal shapes, respectively. Both structures, representing the doublet state, indicate that the bipyramidal configuration (Figure 1a) is more stable in the gas phase compared to the trapezoidal shape (Figure 1b), with an energy difference of 1.38 eV. The bipyramidal Rh_5_ nanoclusters in Figure 1a deviate from perfect D3h symmetry, featuring equatorial Rh atoms forming a triangular ring and axial Rh atoms located above and below the ring. Notably, the equatorial atoms exhibit inequivalence, with three bonds shaping the triangle (*d*_1_), measuring 2.48 Å, while the remaining bond (*d*_4_) is 2.50 Å. The bonds *d*_2_ and *d*_3_, formed by axial Ag with the three equatorial sites measure 2.47 Å for both (see Table 1 for detailed information). The unpaired electron in the Rh_5_ doublet state (with S = ½), as depicted in Figure 1c, primarily localises on the two axial Rh atoms, resembling the charge distribution observed in Cu_5_ [53] and Ag_5_ nanoclusters [33]. According to the density of states analysis in Figure 1, the calculated band gaps of bipyramidal and trapezoidal Rh_5_ nanoclusters are 1.15 eV and 0.57 eV, respectively.

### 3.2. Bipyramidal Rh_5_ Nanocluster Loaded on TiO_2_

**Figure 2:**
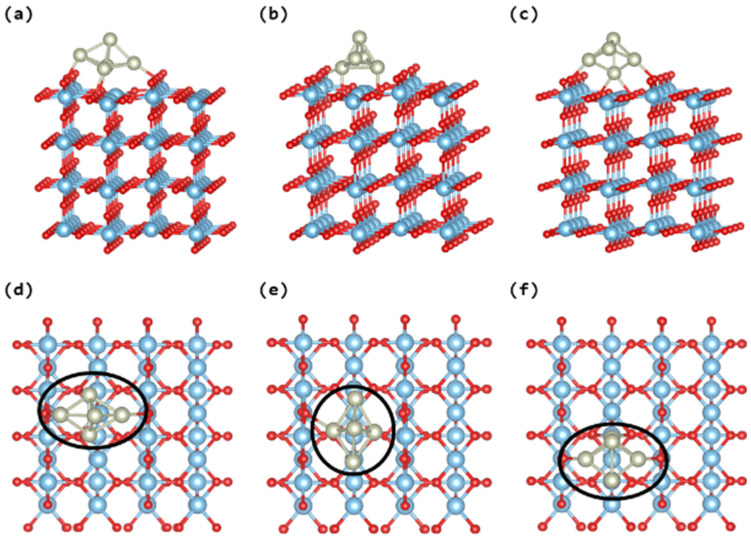
Different adsorption configurations of bipyramidal Rh_5_ nanoclusters at TiO_2_ rutile (110) surface; structures presented in (**a**–**c**) showing the lateral views. While structures presented in (**d**–**f**) are showing the top views. The Rh, Ti, and O atoms are represented by the silver, blue, and red balls, respectively.

As part of a benchmark analysis, we computed the electronic density of states for the pristine TiO_2_ rutile (110), as shown in Figure S1. Our calculations yielded an estimated band gap value of approximately 3.2 eV, aligning well with experimental findings [12]. After examining the geometrical and electronic characteristics of bare Rh_5_ nanoclusters, our focus shifted to an investigation of the corresponding attributes in Rh_5_ nanoclusters adsorbed into both pristine and reduced TiO_2_ (110) surfaces. Three distinct adsorption sites of the bipyramidal Rh_5_ nanocluster on TiO_2_ are scrutinized, as depicted in Figure 2. Notably, the configuration illustrated in Figure 2a demonstrates superior stability, evidenced by an adsorption energy of −5.28 eV in comparison to the other two configurations, with an average Rh-O bond length of 2.10 Å (see Table 2). Conversely, the structure presented in Figure 2b exhibits the least stability, marked by an adsorption energy of −4.78 eV. The configuration in Figure 2c manifests a metastable state with an adsorption energy of −4.84 eV. The discerned disparities in stability may be ascribed to the nature of the Rh-O bonds; specifically, in the most stable configuration (Figure 2a), four Rh atoms are bonded to four O atoms, whereas in the remaining two structures, only three Rh atoms form bonds with three O atoms.

**Figure 3:**
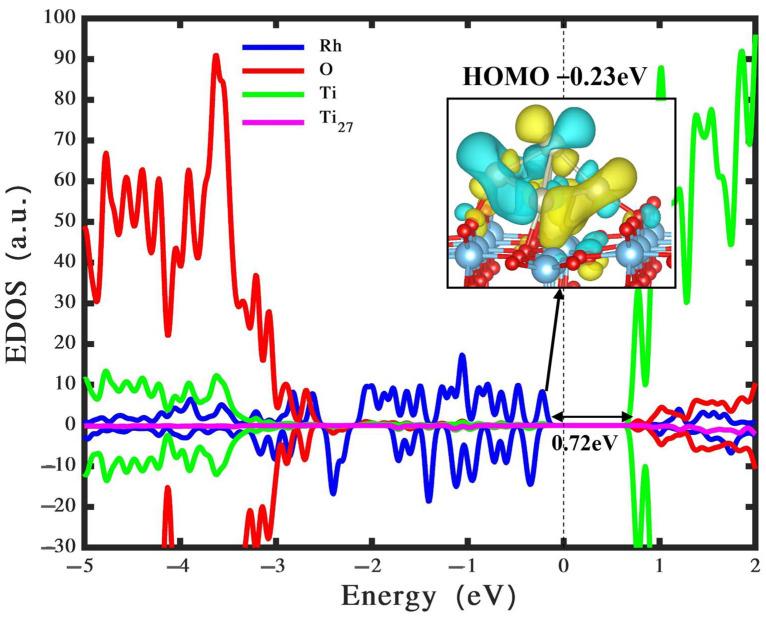
Density of states and wavefunction of bipyramidal Rh_5_ nanocluster loaded on perfect TiO_2_ rutile (110) surface. The states associated with Ti, O, Rh atoms, and Ti_27_ atom are depicted by the green, red, blue, and pink colours, respectively. The Fermi energy level is indicated by the black vertical line. The reference colours yellow and blue for isosurfaces symbolize the positive and negative stages of wave functions, respectively. It’s important to note that these reference colours are consistently used for all wavefunction plots in the following figures.

Additionally, to enhance our understanding of the observed adsorption patterns, we focus on the analysis of charge transfer. In the case of the most stable configuration, the Rh_5_ nanocluster exhibits a charge transfer of approximately +0.6 e^−^ to TiO_2_. This observed electron transfer implies an oxidation state for the Rh_5_ nanocluster, corroborating findings from previous studies [51,54,55]. To evaluate the influence of the Rh_5_ nanocluster on the electronic structures of the TiO_2_ rutile (110) surface, we conducted density of states calculations applying the HSE06 functional and wavefunction computations for the most stable configuration, as depicted in Figure 2a and presented in Figure 3. Our findings indicate that incorporating a bipyramidal Rh_5_ nanocluster into the TiO_2_ rutile (110) surface results in the creation of mid-gap states in the band gap.

As an illustration, the highest occupied molecular orbital (HOMO) exhibits a high-energy state situated at −0.23 eV, roughly 0.72 eV below the CB edge. The introduction of mid-gap states is a result of charge transfer from the Rh_5_ nanocluster to the TiO_2_ surface. These intermediary states play a pivotal role in absorbing photons within the visible and UV regions. Furthermore, the deposition of the Rh_5_ nanocluster on TiO_2_ results in the repopulation of the CB, initiating a manifestation of metallic characteristics within the system. Similar findings have been documented for a TiO_2_ system when exposed to Ag_3_ and Ag_5_ clusters [56]. In the context of the visible-light spectrum, it becomes apparent that mid-gap states can accept electrons from the VB. The energetic nature of visible-light irradiation facilitates electron transfer due to the diminished energy separation between intra-gap states and the VB. This electron transition potentially contributes to the augmentation of photocatalytic hydrogen production [57]. For example, Wang et al. [58] experimentally reported that the photocatalytic activity for hydrogen evolution using Rh-doped rutile demonstrated an approximate fiftyfold increase in efficiency compared to that observed with Rh-doped anatase powders.

### 3.3. Trapezoidal Rh_5_ Nanocluster Loaded on TiO_2_

**Figure 4:**
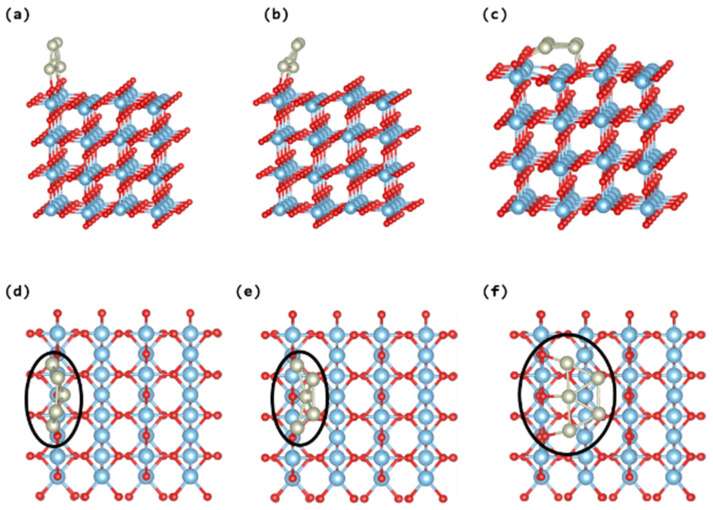
Three adsorption sites of trapezoidal Rh_5_ nanoclusters on TiO_2_ rutile (110) surface; structures presented in (**a**) upstanding Rh_5_, (**b**) titled Rh_5_, and (**c**) lying-down Rh_5_ showing the lateral views. While structures presented in (**d**–**f**) are showing the top views. The Rh, Ti, and O atoms are represented by the silver, blue, and red circles, respectively.

This investigation involves the computational simulation of three distinct adsorption configurations of trapezoidal Rh_5_ nanoclusters on the TiO_2_ rutile (110) surface, specifically adopting upstanding, tilted, and lying-down orientations, as illustrated in Figure 4. Analysis of the simulation reveals notable distortions in the upstanding and tilted Rh_5_ nanoclusters (see Figure 4a,d and Figure 4b,e) upon their adsorption onto the TiO_2_ surface, leading to diminished stability, characterised by an average Rh-O bond length of approximately 2.02 Å. A higher adsorption energy is discerned in the case of a slight tilt in the Rh_5_ nanocluster towards the TiO_2_ surface, amounting to approximately 0.01 eV. Conversely, when the Rh_5_ nanocluster assumes a parallel orientation to the TiO_2_ surface (depicted in Figure 4c,f), a considerably higher adsorption energy of approximately −6.46 eV is observed, indicative of enhanced stability in comparison to the upstanding and tilted configurations. This trend mirrors findings from prior DFT studies on trapezoidal Ag_5_ and Cu_5_ adsorbed on TiO_2_ rutile (110) [33,53]. Additionally, a pronounced distortion is evident on the TiO_2_ surface directly beneath the loaded Rh_5_ nanocluster. Table 3 provides a comparative analysis of the adsorption energies and charges associated with the various Rh_5_ nanocluster structures depicted in Figure 4.

**Figure 5:**
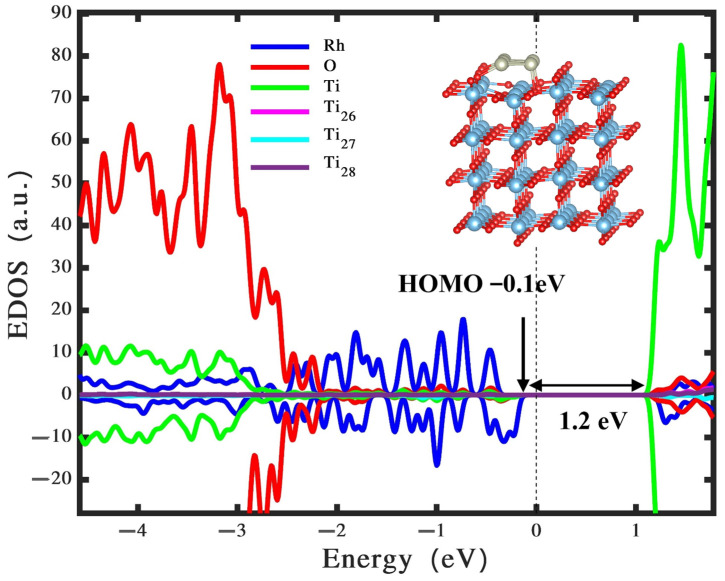
Density of states of the trapezoidal Rh_5_ nanocluster loaded on perfect TiO_2_ (110) surface. The states suited on Ti, O, Rh, Ti_26_, Ti_27_, and Ti_28_ atoms are represented by the green, red, blue, pink, cyan, and purple colours.

The electronic characteristics of the most stable structure of trapezoidal Rh_5_@TiO_2_ are subjected to an in-depth analysis through the density of states and analysis of Bader charge, as presented in Figure 4 and Table 3, respectively. Bader charge analysis reveals that all trapezoidal Rh_5_ nanoclusters exhibit electron donation to the TiO_2_ surface, inducing oxidation. Interestingly, a noticeable correlation is observed, where less charge transfer from the Rh_5_ nanocluster to the catalyst correlates with higher stability, while augmented charge transfer corresponds to less stability. This trend contrasts with the behaviour noted in the loading of Ag_5_ clusters on the TiO_2_ rutile (110) surface [33]. Furthermore, the adsorption of the trapezoidal Rh_5_ nanocluster induces notable alterations in the electronic characteristics of the pristine TiO_2_, generating mid-gap states within the band gap, as illustrated in Figure 5. The density of states analysis delineates that the HOMO state of the Rh_5_ nanocluster is situated approximately 1.2 eV below the CB. To conclude this section, the simulation results show that the bipyramidal Rh_5_ nanocluster exhibits superior efficacy in enhancing the photocatalytic activity of TiO_2_ rutile (110) compared to the trapezoidal Rh_5_ nanocluster. This is substantiated by the energy difference, with the most stable configuration of the bipyramidal Rh_5_ nanocluster registering a –0.2 eV reduction compared to the most stable structure of the trapezoidal Rh_5_ nanocluster.

### 3.4. Bipyramidal Rh_5_ Nanocluster Loaded on Defective TiO_2_

To investigate the influence of the Rh_5_ nanocluster on the generation of an oxygen vacancy on the TiO_2_ rutile (110) surface, we initially present results related to defective TiO_2_ rutile (110). In our prior DFT calculations [33], it has been demonstrated that the formation energy of a surface oxygen vacancy on pristine TiO_2_ rutile (110) is lower than that of the subsurface by approximately 0.6 eV (see Figure S2 and Table S1), aligning with previous studies [59,60]. Subsequently, with reference to the most stable configuration of Rh_5_ loaded on TiO_2_, as depicted in Figure 2a, an exploration into the impact on the photocatalytic activity concerning surface oxygen vacancy was conducted. The investigation reveals that the introduction of the Rh_5_ nanocluster stabilises TiO_2_ rutile (110), leading to an elevation in the formation energy of both surface and subsurface oxygen vacancies by 0.44 eV and 0.17 eV, respectively (see Figure S3 and Table S1 for further comparative analysis).

**Figure 6:**
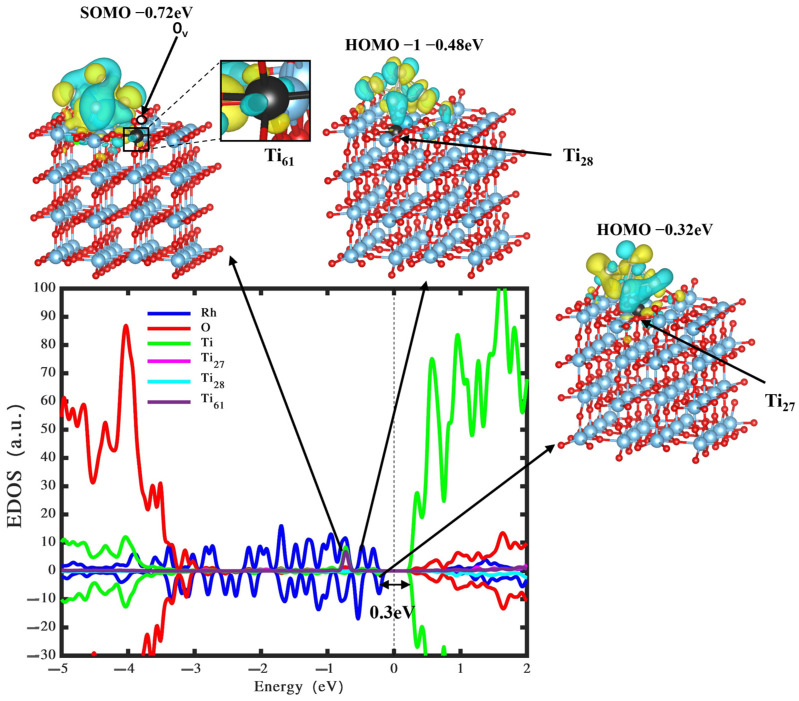
Density of states and wavefunction of the bipyramidal Rh_5_ nanocluster loaded on reduced TiO_2_ (110) surface. The green, red, blue, pink, cyan, and purple represent the states located on Ti, O, Rh, Ti_27_, Ti_28_, and Ti_61_ atoms.

To explore the electronic characteristics of a Rh_5_ nanocluster adsorbed onto reduced TiO_2_ rutile (110), we conducted density of states and wavefunction calculations, as shown in Figure 6. The figure indicates that the combined presence of the Rh_5_ nanocluster and an oxygen vacancy introduces additional gap states. Notably, the CB edge undergoes a significant downward shift towards lower energy levels, resulting in an elevated energy HOMO state of the Rh_5_ nanocluster by approximately 0.3 eV from its position. Remarkably, the state appearing at –0.72 eV corresponds to the singly occupied molecular orbital (SOMO), situated on a Ti_61_ atom on the surface of TiO_2_, exhibiting an electron gain of approximately 0.3 e^−^ and giving rise to a polaronic state (as depicted by SOMO in Figure 6). Furthermore, our investigation shows an electron donation from the Rh_5_ nanocluster to the material amounting to +0.3 e^−^, representing a reduction of half compared to the configuration without the oxygen vacancy (i.e., the configuration illustrated in Figure 2a). The formation of the polaronic state is identified as a significant factor contributing to the absorption of visible-light photons [61]. In summary, the reciprocal presence of the Rh_5_ nanocluster and the oxygen vacancy synergistically enhances the photocatalytic activity of the substrate. Consequently, both the Rh_5_ nanocluster and the oxygen vacancy emerge as potential catalysts for water splitting, offering promising insights for the systematic design of highly efficient photocatalysts dedicated to photocatalytic hydrogen generation.

## 4. Concluding Remarks

This article delves into the investigation of geometrical stabilities and electronic characteristics of Rh_5_ nanoclusters on the TiO_2_ rutile (110) surface, aiming for potential applications in green hydrogen production through photocatalytic water splitting. The research is motivated by the need for efficient and sustainable energy sources, particularly focusing on enhancing the photocatalytic performance of TiO_2_, a well-established photocatalyst. By employing DFT with the DFT-D3 technique and the HSE06 hybrid functional, we conducted a comprehensive examination of the adsorption behaviour, electronic structures, and charge transfer dynamics of Rh_5_ nanoclusters on TiO_2_ surfaces. The computational analysis explored the stability of Rh_5_ nanoclusters on both pristine and reduced TiO_2_ surfaces, along with their influence on oxygen vacancy formation. The results indicate that trapezoidal Rh_5_ nanoclusters exhibit superior stability and adsorption energy compared to bipyramidal structures when deposited on TiO_2_ rutile (110). The most stable adsorption structures of the bipyramidal Rh_5_ nanocluster resulted in a charge transfer of approximately +0.6 e^−^ to TiO_2_, inducing oxidation of the nanocluster. Electronic structure analysis reveals the generation of intra-gap states in the band gap of TiO_2_ upon Rh_5_ nanocluster deposition, suggesting potential implications for visible and ultraviolet photon absorption.

Additionally, this study investigates the interaction of Rh_5_ nanoclusters with defective TiO_2_ surfaces, with a specific focus on the oxygen vacancies’ evolution. The results indicate that the existence of Rh_5_ nanoclusters stabilizes TiO_2_ and increases the oxygen vacancies’ formation energy, hinting at a potential role in enhancing photocatalytic activity. To summarise, this theoretical investigation offers valuable insights into the potential of Rh_5_ nanoclusters as efficient cocatalysts for TiO_2_-based photocatalytic systems. The results propose that the bipyramidal configuration of Rh_5_ nanoclusters, when appropriately adsorbed on TiO_2_ rutile (110), may contribute to enhanced photocatalytic performance, providing opportunities for the rational design of highly efficient photocatalysts for green hydrogen production. The HOMO state being located at 0.72 eV below the CB edge (see Figure 3), which possesses high energy, can significantly benefit photocatalytic water splitting for green hydrogen production. This positioning of the HOMO level enhances the ability of the photocatalyst to transfer electrons effectively. During water splitting, electrons in the HOMO can be excited to the CB, leaving holes in the HOMO. These holes can then participate in the oxidation of water to produce oxygen. The excited electrons in the CB can reduce protons in water, generating hydrogen. Therefore, the position of the HOMO level is crucial for efficient photocatalytic activity, influencing the HER in water splitting processes. The observed effects on oxygen vacancy formation further underscore the intricate interplay between metal nanoclusters and the semiconductor surface, paving the way for future experimental validations and practical applications in sustainable energy production.

## Figures and Tables

**Table 1:** Bond lengths of the different Rh_5_ nanoclusters shown in Figure 1a,b.

Bond Length (Å)	Bipyramidal Rh_5_	Trapezoidal Rh_5_
*d*_1_	2.48	2.44
*d*_2_	2.47	2.40
*d*_3_	2.47	2.44
*d*_4_	2.50	2.53
*d*_5_	2.51	-

**Table 2:** DFT + U calculated adsorption energies (*E_ads_*) and Bader charge distributions on adsorbed bipyramidal Rh_5_ nanoclusters shown in Figure 2.

Structure	Figure 2a	Figure 2b	Figure 2c
*E_ads_* (eV)	−5.28	−4.78	−4.84
Charge on Rh_5_ (e^−^)	+0.60	+0.85	+0.80

**Table 3:** DFT + U calculated adsorption energies (*E_ads_*) and Bader charge distributions on adsorbed trapezoidal Rh_5_ nanoclusters shown in Figure 4.

Structure	Figure 4a	Figure 4b	Figure 4c
*E_ads_* (eV)	−3.90	−3.91	−6.46
Charge on Rh_5_ (e^−^)	+0.79	+0.80	+0.72

## Data Availability

Data are contained within the article.

## Supplementary Materials

The following supporting information can be downloaded at: https://www.mdpi.com/article/10.3390/nano14020191/s1, Figure S1: Density of states of pristine rutile TiO_2_ (110). The green and red curves show the electronic density of states on titanium and oxygen atoms, respectively. The black vertical dashed line shows the Fermi energy level. Reproduced from our previous calculations [33]; Figure S2: Oxygen vacancy formation at (a) surface and (b) subsurface locations of TiO_2_ rutile (110). The black circles represent the oxygen vacancy position. Reproduced from our previous calculations [33]; Figure S3: Oxygen vacancy formation at (a) surface and (b) subsurface locations of Rh_5_@TiO_2_ rutile (110). The black circles represent the oxygen vacancy position; Table S1: Comparisons of formation energies of oxygen vacancy.
