# Supplementary material for: Geometrical Stabilities and Electronic Structures of Rh5 Nanoclusters on Rutile TiO2 (110) for Green Hydrogen Production
Source: Nanomaterials (Basel). 2024 Jan 15;14(2):191. doi: 10.3390/nano14020191 (PMC10819950; doi:10.3390/nano14020191)
Supplement: Supplementary file 1 [file nanomaterials-14-00191-s001.zip › nanomaterials-2822460-supplementary.pdf]

Geometrical Stabilities and Electronic Structures of Rh_5 Nanocluster on Rutile TiO_2 (110) for Green Hydrogen Production

Moteb Alotaibi

Department of Physics, College of Science and Humanities in Al-Kharj, Prince Sattam Bin Abdulaziz University, Al-Kharj 11942, Saudi Arabia; mot.alotaibi@psau.edu.sa

1. TiO_2

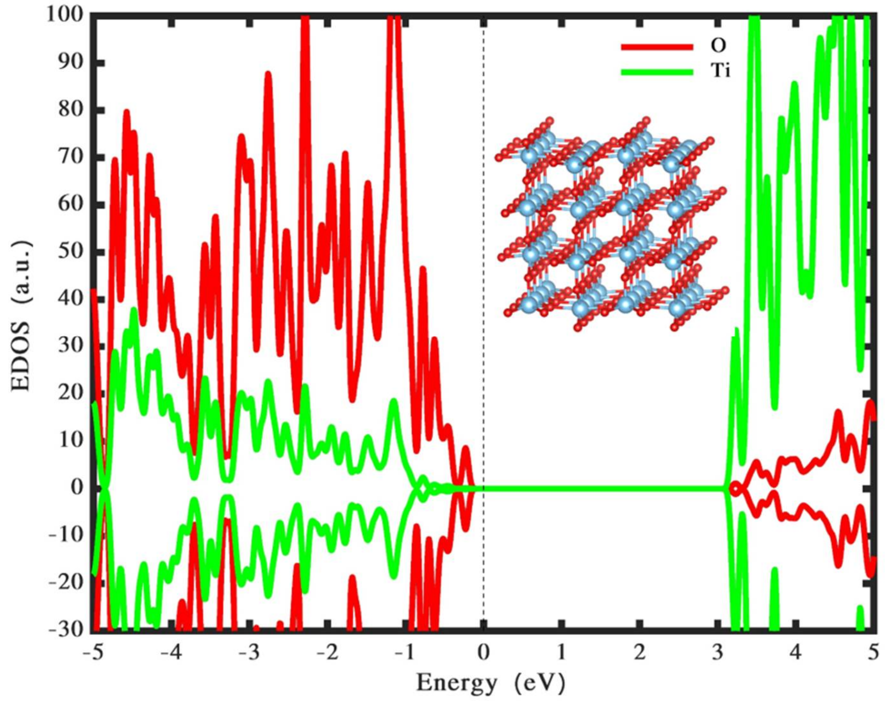

Figure S1 Density of states of pristine rutile TiO_2 (110). The green and red curves show the electronic density of states on titanium and oxygen atoms, respectively. The black vertical dashed line shows the Fermi energy level. Reproduced from our previous calculations [1].

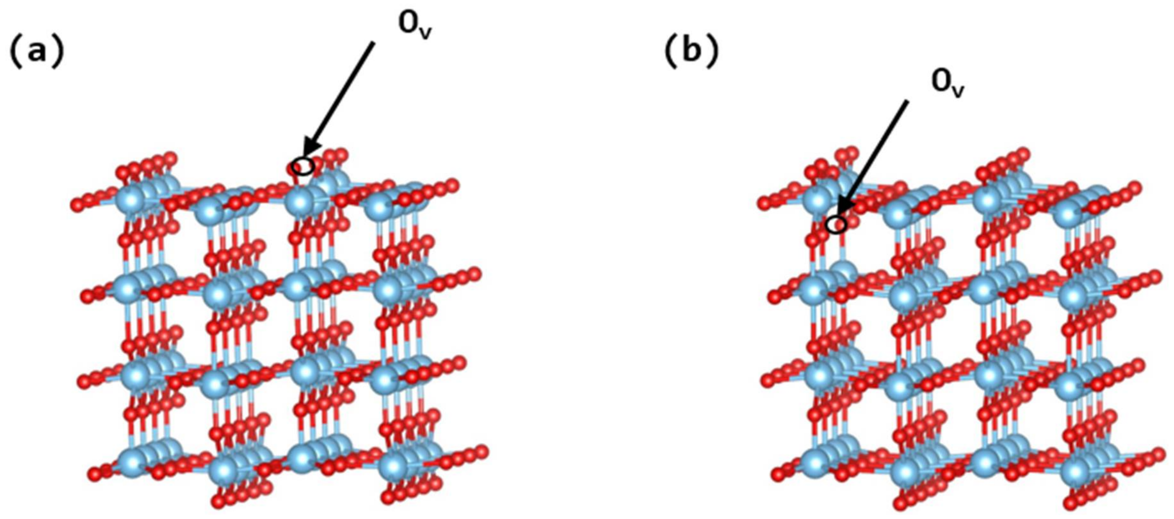

Figure S2 Oxygen vacancy formation at (a) surface and (b) subsurface locations of TiO_2 rutile (110). The black circles represent the oxygen vacancy position. Reproduced from our previous calculations [1].

2. **Rh_5@reduced TiO_2**

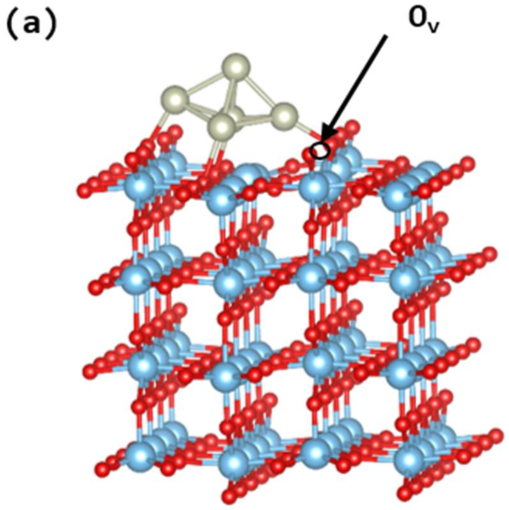

Figure S3 Oxygen vacancy formation at (a) surface and (b) subsurface locations of Rh_5@TiO_2 rutile (110). The black circles represent the oxygen vacancy position.

Table S1 Comparisons of formation energies of oxygen vacancy.

**Structure**	**(a) In Figure S2**	**(b) In Figure S2**	**(a) In Figure S3**	**(b) In Figure S3**
**Formation energy of oxygen vacancy (eV)**	4.07	4.65	4.50	4.82

Reference

[1] M. Alotaibi, Q. Wu, and C. Lambert, “Computational Studies of Ag 5 Atomic Quantum Clusters Deposited on Anatase and Rutile TiO 2 Surfaces,” *Appl Surf Sci*, p. 156054, 2022, doi: 10.1016/j.apsusc.2022.156054.
